# Effects of “Thursdays at the Museum” at the Montreal Museum of Fine Arts on the mental and physical health of older community dwellers: the art-health randomized clinical trial protocol

**DOI:** 10.1186/s13063-020-04625-3

**Published:** 2020-08-12

**Authors:** Olivier Beauchet, Liam Cooper-Brown, Yoko Hayashi, Kevin Galery, Christine Vilcocq, Thomas Bastien

**Affiliations:** 1 Department of Medicine, Division of Geriatric Medicine, Sir Mortimer B. Davis Jewish General Hospital and Lady Davis Institute for Medical Research, McGill University, 3755 chemin de la Côte-Sainte-Catherine, Montréal, Quebec H3T 1E2 Canada; 2 Dr. Joseph Kaufmann Chair in Geriatric Medicine, Faculty of Medicine, McGill University, Montreal, Quebec Canada; 3 Centre of Excellence on Longevity of the McGill Integrated University Health Network, Montreal, Quebec Canada; 4 Lee Kong Chian School of Medicine, Nanyang Technological University, Singapore City, Singapore; 5 Faculty of Informatics for Arts Department of Information Expression, Shobi University, Kawagoe, Japan; 6 Education and Wellness Department of the Montreal Museum of Fine Arts, Montreal, Quebec Canada

**Keywords:** Older adults, Randomized controlled trial, Art, Museum, Well-being, Quality of life, Health

## Abstract

**Background:**

Recently, we demonstrated that the Montreal Museum of Fine Arts’ (MMFA) participatory art-based activity, known as “Thursdays at the Museum,” improved the well-being, quality of life, and physical health (i.e., frailty) of older community dwellers by using a pre-post intervention, single arm, prospective and longitudinal experimental design. The present randomized clinical trial (RCT), known as the Art-Health RCT (A-Health RCT), aims to compare changes in well-being, quality of life, frailty, and physiological measures in older community dwellers who participate in “Thursdays at the Museum” (intervention group) and in their counterparts who do not participate in this art-based activity (control group).

**Methods/design:**

The current unicenter, randomized, clinical, controlled, comparative trial recruits 150 older community dwellers to two parallel arms (75 participants in the intervention group and 75 participants in the control group). The intervention is a 3-month cycle of weekly “Thursdays at the Museum,” which are structured 2-h-long art-based workshops performed in a group setting at the MMFA. The control group is composed of participants who do not take part in art-based activities, receive their usual health and/or social services, and commit to report any other activity practiced during the same time. Assessments of the primary outcome (well-being) and the secondary outcomes (quality of life, frailty, and physiological measures including heart rate, daily step count, sleep duration, and its phases) are performed on six occasions: at baseline, at the beginning of the second and third months, at the end of the third month, as well as 6 and 12 months after the last workshop. Statistical analyses are performed with the intention to treat and per protocol. Comparisons of changes in outcome measures between intervention and control groups use repeated measures tests.

**Discussion:**

Art-based activities carried out at museums have been receiving increased interest from researchers and policy-makers because of their benefits to mental and physical health. There are few robust studies, such as RCTs, that focus on older community dwellers or assess the efficacy of these participatory museum activities. The A-Health RCT study provides an opportunity to confirm the benefits of a participatory art-based museum activity on the elderly population and to show the key role played by museums in public health promotion.

**Trial registration:**

NCT03679715; Title: A-Health RCT: Effects of Participatory Art-Based Activity on Health of Older Community Dwellers; First posted date: September 20, 2018; prospectively registered.

## Background

Participation in creative art activities has been receiving increased interest in the past decade [1, 2]. It has been shown that participatory art-based activities may improve aspects of mental health such as positive emotions and self-esteem [3–6]. Art-based activities help patients, regardless of their disease, to build a sense of self, transforming the illness experience into a positive experience and improving patients’ well-being and quality of life [5–8]. Well-being, defined as a good or satisfactory condition of existence, quality of life, and health are closely related, and this relationship is important at older ages [9, 10]. Well-being is positively associated with quality of life and physical health benefits, including a decreased risk for disease, speedier disease recovery, and increased longevity [6–13]. In parallel, it has been found that art-based activities are positively associated with numerous aspects of individuals’ physical health, like a better immune system response and slower disease progression, with these effects being related to well-being improvement [3, 6–8]. These mental and physical health benefits suggest that art-based activities may be effective interventions for frailty prevention in older community-dwellers. The elderly population’s health condition is heterogeneous due to the variously combined effects of physiological aging-associated decline and morbidities [14]. Frailty, which is used to classify the health condition of older adults and their ability to respond to an intervention, is a condition of vulnerability related to an accumulation of morbidities and exposing individuals to incident adverse health events, which increase health and social costs [14–16]. The frailty process may be prevented or even reversed, especially at its onset [15]. Older individuals with mild frailty benefit the most from interventions that can promote health and prevent frailty from worsening [15]. Thus, it seems that older adults with frailty—especially those at its onset—will particularly benefit from engaging in art-based activities. There are few robust experimental studies, such as randomized clinical trials (RCT) that regard the effects of participatory art-based activities on mental and physical health and, thus, more information is needed, especially about older adults and those with frailty.

Museums have designed their spaces to create participatory art-based activity programs that can be carried out in a pleasant environment [8, 17–20]. For instance, in 2015, the Montreal Museum of Fine Arts (MMFA) developed “Thursdays at the Museum,” which is a participatory art-based activity performed by older community dwellers who live in Montreal creating art in group settings. In 2018, the effects of this participatory art-based activity on the well-being, quality of life, and frailty (i.e., physical health) of older community dwellers were examined [21]. With a pre-post intervention, single arm, prospective, and longitudinal design, we showed that the MMFA’s participatory art-based activity improved the well-being, quality of life, and frailty of older community dwellers in Montreal. Each outcome’s improvement evolved differently over the 3-month cycle of weekly art-based activities. Well-being was greater at the end of each workshop when compared to the beginning, with a similar magnitude of change throughout the cycle. In contrast, quality of life improved gradually over the course of the cycle. Finally, when comparing the cycle’s end to its beginning, the proportion of vigorous participants was greater, whereas the proportion of mildly frail participants was smaller. A major limitation of our first study was its design, as the gold standard to examine the effects of an intervention is a RCT in parallel groups.

Findings from our first study suggest that “Thursdays at the Museum” may improve older adults’ mental (i.e., well-being and quality of life) and physical (i.e., frailty) health. The relationship between well-being, quality of life, and physical health is complex. Previous studies, including our first study, suggested the existence of a chronological sequence of benefits: positive experiences induced by art-based activities improve well-being, which improves quality of life and, finally, physical health [6–13, 21]. This sequence may explain the changes in outcomes over time reported in our first study. However, the changes in well-being, quality of life, and physical health that older community dwellers experience over time and independently of any intervention are sparsely known. These aging-related changes may influence the effects specific to “Thursdays at the Museum.” We hypothesize that changes in well-being, quality of life, and frailty due to older community dwellers’ participation in “Thursdays at the Museum” (1) each have not the same profile of evolution over time; (2) are greater than those observed with aging (i.e., experienced by the older adults with or without morbidities that make up the control group); (3) are improvements of well-being, quality of life, and frailty; and (4) may persist after the discontinuation of “Thursdays at the Museum.”

## Objective

This study aims to compare changes in well-being, quality of life, physical health (i.e., frailty), and physiological measures (heart rate, daily step count, sleep duration, and its phases) in older community dwellers participating in a 3-month cycle of “Thursdays at the Museum” (intervention group) to the corresponding changes in their randomized counterparts who do not participate in this MMFA art-based activity (control group).

## Methods

### Design

The design is a unicenter (MMFA; Montreal, Quebec, Canada), clinical, randomized, controlled trial with two parallel arms (intervention and control groups) that is comparative (comparison of intervention and control groups). The participants in the intervention group participate in “Thursdays at the Museum.” The control group is composed of older community dwellers who do not participate in “Thursdays at the Museum.” They are not deprived of any indicated health or social services, and they continue to be followed by their health and social professionals. They commit to withhold participation in art-related interventions for the study period and to report any other activity practiced during the same time. Due to the nature of the intervention, participants cannot be blind to it. The principal investigator, his representatives and all staff members involved in the RCT phases (i.e., recruitment, assessment, and follow-up of participants) are blinded to the allocation of the intervention, except one staff member who is not involved in these RCT phases. Blinding is also applied after data collection (from data analysis to the reporting of results).

### Participants

#### Selection criteria

Being 65 and over, understanding and writing French or English, having Internet access at their place of living and an electronic device, having a life expectancy over 15 months, and providing and signing a written informed consent to participate in the RCT are the criteria for inclusion. The principal investigator or representatives will provide the individual with written and oral information about the study and any special considerations in the informed consent form and the consenting process in a language they can understand. The individual then signs an informed consent form. Life expectancy is estimated with a free software using socio-demographic, cardio-vascular risk factors, physical activity, and income characteristics (https://www.blueprintincome.com/tools/life-expectancy-calculator-how-long-will-i-live/). The exclusion criteria are concomitant participation in another experimental study regardless of its type; major disabilities like deafness, blindness, and gait disorders; and major neurocognitive impairment. This information is collected during the recruitment process by phone call.

#### Setting and recruitment

**Fig. 1:**
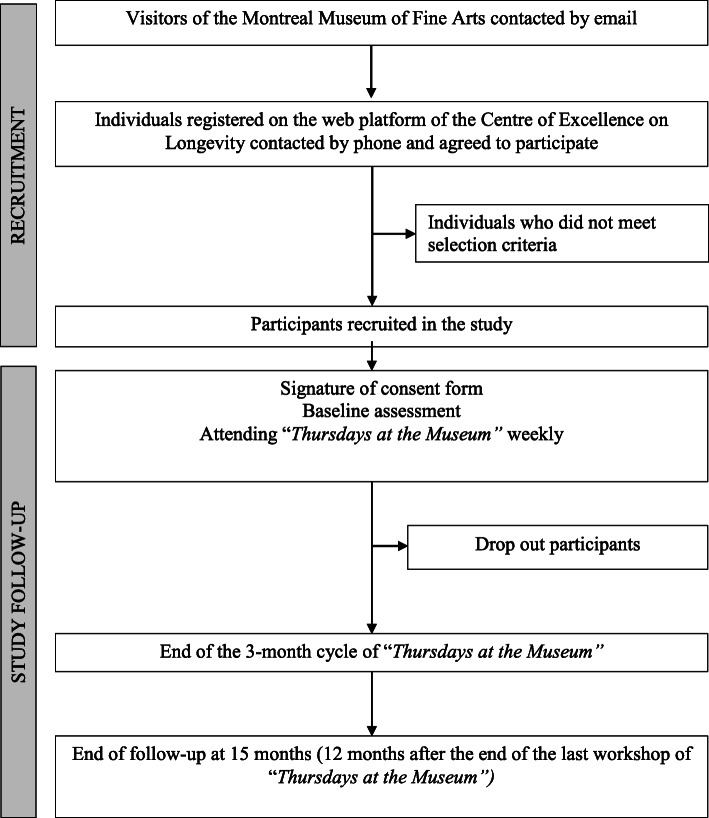
Flow chart of screening, recruitment, and follow-up

The Art-Health RCT (A-Health RCT) is an ongoing study carried out at the MMFA (Quebec, Canada). The participant recruitment and follow-up processes are shown in Fig. 1. Museum visitors aged 65 and over are informed by an email from the MMFA that an experimental study on the effects of “Thursdays at the Museum” is ongoing at the MMFA. Individuals who are interested in participating register on the web platform of the Centre of Excellence on Longevity (CEEXLO) using a link provided at the end of the MMFA’s email. After registration, they are contacted by phone by a representative of the principal investigator to assess the selection criteria. If they meet all criteria and agree to participate in the RCT, they are recruited. Information on non-included participants and on those who withdraw their consent and/or drop out during the 15-month period of follow-up is recorded.

#### Randomization

After the baseline assessment, participants are randomly allocated to intervention and control groups using block randomization with a 1:1 ratio. The randomization list is established with the N’Query randomization software by the project manager, who is the only person able to identify participants based on their code. All investigators are blind to randomization. The project manager is not involved in the recruitment nor the follow-up of participants. He conducts the randomization process and informs the participants of to which group they are assigned. Randomization is performed the week before the first workshop.

Furthermore, a subset of participants is offered a Fitbit Alta HR (Fitbit, Inc.; San Francisco, CA, USA) to record physiological measures (i.e., heart rate, daily step count, sleep duration, and its phases). The subset is limited to 30 intervention group and 30 control group participants, determined in a second randomization that keeps the subsets as similar as possible to control for selection bias. This second randomization adopts the process used during the first randomization and the same project manager oversees it to ensure that all investigators remain blind. After the second randomization is completed, the project manager in charge of randomization provides selected participants with a Fitbit device, an assessment schedule and instructions. The project manager also installs the Fitbit application on participants’ portable electronic devices and creates their individual accounts with coded usernames.

### Intervention

“Thursdays at the Museum” is the participatory art-based activity examined in this RCT. This hands-on activity consists of workshops during which 30 individuals create art as a group. Study participants assigned to partake in workshops are separated into two equal groups, with one group meeting on Tuesdays and the other on Wednesdays. These participants meet weekly in a room at the MMFA dedicated to the 2-h workshop. The total duration of a “Thursdays at the Museum” cycle is 3 months. Each month, a different topic is explored through 4 consecutive workshops. The topics are abstract painting, live model drawing, and stained-glass painting. Each workshop is supervised by two cultural mediators. They manage the interactive hands-on art activities, with special attention given to creativity, handcrafting techniques, and fine motor skills. Each workshop is standardized, consisting of three consecutive phases: presentation of the art activity’s objective, choice of medium, and communication of technical advice to participants.

### Assessment

#### Baseline assessment

The baseline assessment is performed with all participants (intervention and control) during a first meeting in a dedicated room at the MMFA. It is a standardized interview performed by a representative of the principal investigator and is carried out before (M0) the first workshop, at the time when participants sign the written consent form. All measurement types and their times of use are shown in Table 1. All questionnaires and scales used are reliable and previously validated. No feedback on assessment results is shared with participants.

**Table 1:** Assessed domains, instruments and time points

Domains	Instruments	Time points								
		Baseline (M0)		Follow-up						
		Before first workshop	After first workshop	M1		M2		M3		M9 and M15
				Before fifth workshop	After fifth workshop	Before ninth workshop	After ninth workshop	Before twelfth workshop	After twelfth workshop	
Ability to use a computer	CPQ	X								
Socio-demographic characteristics		X								X
Frail condition	CESAM	X		X		X			X	X
Well-being	WEMWBS	X	X	X	X	X	X	X	X	X
Quality of life	EQ-5D	X		X		X			X	X
Physiological measures		X		X		X			X	X
Physician consultation Emergency room visit and hospitalization		X		X		X			X	X

*M* month, *M0* baseline assessment, *M1* first month, *M2* second month, *M3* thrid month, *M9* 6 months after the end of the intervention, *M15* 12 months after the end of the intervention, *CPQ* computer proficiency questionnaire, *CESAM* Centre of Excellence Self-AdMinistered questionnaire, *WEMWBS* Warwick-Edinburgh Mental Well-being Scale, *EQ-5D* EuroQol-5D

First, the computer proficiency questionnaire (CPQ), which assesses respondents’ ability to use a computer, is performed in paper format [22]. Its scores range from 5 (i.e., never tried) to 30 (i.e., can do very easily). Second, socio-demographic characteristics including age, sex, and place of living are recorded using the CEEXLO web platform. Third, frailty is assessed with the digital short form of a self-administered questionnaire: The Centre of Excellence Self-AdMinistered questionnaire (CESAM, Table 2) [23]. This questionnaire is filled out by participants under the supervision of the principal investigator’s representatives. The 20 items that make up the CESAM explore several domains, including weight loss and its magnitude; polypharmacy, defined as a number of therapeutic classes taken on a daily basis ≥ 5; vision, hearing, and memory problems; use of home support (i.e., family, friend, and/or professional); the 6-item activities of daily living (ADL) scale and the 4-item instrumental activities of daily living (IADL) scale [24]; mood, assessed using the question, “How do you feel today?” with three possible answers (happy, unhappy, and neither one nor the other); practice of regular physical activity (e.g., walking and bicycle), defined as at least 1 h per week in the past month; and history of falls in the past 12 months. Each CESAM item is close-ended (i.e., yes, no, or choose a specific answer). Automatically, two scores are calculated: (1) a frailty score, ranging from 0 (i.e., best health and functional condition) to 18 (i.e., worst health and functional condition) and (2) a categorization of frailty into one of four stages (vigorous with a score between 0 and 3, mild frailty with a score between 4 and 7, moderate frailty with a score between 8 and 12, and severe frailty with a score above 12). Fourth, EuroQol-5D (EQ-5D) is used to assess participants’ quality of life [25]. This assessment is comprised of two parts. The first is a questionnaire containing five questions coded from 1 (i.e., no issue) to 5 (i.e., worst issue), resulting in total scores ranging from 0 (i.e., no issue) to 25 (i.e., worst issue). The second part of EQ-5D is a visual analog scale (VAS) that explores how good or bad participants perceive their health to be. This scale ranges from 0 (i.e., worst health a participant can imagine) to 100 (i.e., best health a participant can imagine). Fifth, the Warwick-Edinburgh Mental Well-Being Scale (WEMWBS) is used to assess mental well-being [26]. This questionnaire consists of 14 positively worded items with five response categories that cover several aspects of positive mental health. Its scores range from 14 (i.e., none of the time) to 70 (i.e., all the time).

**Table 2:** Self-administered question items and additional questions for follow-up

Item	Answer	
Have you unwillingly lost weight in the past year?	Yes	No
If yes, was the loss of weight above 3 kg/6 lbs?	Yes	No
How many different types of drugs do you take on a daily basis?	- 0	
	- 1–4	
	- 5–9	
	- ≥ 10	
Do you have vision problems?	Yes	No
Do you have hearing problems?	Yes	No
Has someone close to you expressed concern about your memory?	Yes	No
Do you receive home care support?	- Family	
	- Friend	
	- Professional	
If yes, from whom?	Yes	No
Do you need help with your grooming (brushing teeth, hair, shaving, applying make-up)?	Yes	No
Do you need help with bathing or taking a shower?	Yes	No
Do you need assistance when getting dressed?	Yes	No
Do you use mobility aides for walking or transferring (cane, walker, wheelchair)?	Yes	No
Do you need help with your meals: Shopping for food, meal preparation, assistance in eating	Yes	No
Do you need help when using the telephone?	Yes	No
Do you need assistance when taking public transportation?	Yes	No
Do you need help for managing medications on your own?	Yes	No
Do you need help to pay your bills and manage your finances?	Yes	No
Are you incontinent (urine and/or stool)?	Yes	No
How do you feel today?	- Happy	
	- Unhappy	
	- Neither one nor the other	
Do you feel energetic?	Yes	No
Do you do regular physical activity (walking, swimming, cycling, etc.) at least 1 h per week in the past month?	Yes	No
Have you fallen in the past year (at least one fall)?	Yes	No
Additional questions asked to the participants before completing the CESAM at M1, M2, and M3	Yes	No
Since you last filled out this questionnaire, have you visited your doctor?	Yes	No
If yes, was this visit unplanned?	Yes	No
Since you last filled out this questionnaire, have you been hospitalized?	Yes	No
If yes, was this hospitalization unplanned?	Yes	No
Did you visit the emergency room?	Yes	No

Physiological measures including heart rate, number of daily steps, and sleep duration and its phases are recorded with a Fitbit Alta HR. This device is built like a watch and continually records data for periods of 5 consecutive days (2 days before the workshop, the day of the workshop, and 2 days after the workshop). No biological samples are collected in this RCT.

#### Follow-up assessments

In addition to baseline assessment (M0), WEMWBS, CESAM, and EQ-5D are repeated before workshops five (beginning of the second month, M1), nine (beginning of the third month, M2), and twelve (end of the third month, M3) in the intervention and control groups. The week before the first, fifth, ninth, and twelfth workshops, all participants complete questionnaires alone at home using the CEEXLO web platform.

In the intervention group, participants complete the WEMWBS assessments while at the MMFA before and after the first, fifth, ninth, and twelfth workshops. In addition, before CESAM questionnaire completion, four additional questions with binary-coded answers (yes versus no) are asked: (1) Have you seen a physician since the last assessment? (2) If yes, was it a planned or unplanned visit? (3) Have you been hospitalized since the last assessment? (4) If yes, was it a planned or unplanned hospitalization?

Physiological measures are recorded in the intervention and control groups using the same schedule as the previous outcomes with 6 assessment times: M0, M1, M2, M3, as well as 6 and 12 months after the end of the intervention (M9 and M15). All participants who completed the initial 3-month intervention period are reassessed at M9 and M15. This follow-up assessment employs the same questionnaires as during the 3-month intervention. It is performed at participants’ place of living through the CEEXLO web platform with phone support, as needed. We minimize losses to follow-up by scheduling telephone interviews in advance or sending email reminders (for those who agree to this medium of communication).

### Outcomes

The primary outcome is the change in WEMWBS score between the baseline assessment (M0) and the end of the 3-month cycle (M3), calculated using the formula ((score M^*n* + 1^ – score M^*n*^)/((score M^*n* + 1^ + score M^*n*^)/2)) × 100 [21, 26], expressed in percentage. Mean values and standard deviations will be used to define quantitative variables. The secondary outcomes are (1) the changes in WEMWBS scores between M0 and M1, M0 and M2, M0 and M9, and M0 and M15; (2) the mean WEMWBS scores before and after workshops in the intervention group at M0, M1, M2, and M3; (3) the changes in CESAM scores and its distribution of frailty categories (vigorous versus mild, moderate, and severe frailty) between M0 and M1, M0 and M2, M0 and M3, M0 and M9, and M0 and M15 [23]; (4) the change in EQ-5D scores, including VAS scores, between M0 and M1, M0 and M2, M0 and M3, M0 and M9, and M0 and M15 [25]; (5) the frequency of planned and unplanned physician visits, ER visits, and hospital admissions at M1, M2, M3, M9, and M15 [14, 16]; and (6) the changes in mean values of heart rate, daily steps, an sleep duration, and sleep phases, expressed in percentages, between M0 and M1, M0 and M2, and M0 and M3 for the subset of participants with recordings of these physiological measures. All changes will be expressed in percentages as mean values and standard deviations.

The number of workshops attended during the 3-month cycle of intervention is used to assess compliance with the participatory art-based activity. Compliance is defined as having attended at least 10 (83.3%) of the 12 workshops. If a participant attends a workshop for at least 1 h, they are considered to have attended a full workshop. If not, they are recorded as absent.

### Data safety and storage

For the duration of this RCT, the principal investigator (PI) and representatives collect and store personal identifiable information about participants in a digital file for the collection of clinical and physiological data and a paper copy of the signed consent form. Only information needed for the study is collected. The digital information is stored on the secured medical webserver of RUISSS McGill Center of Excellence on Longevity. Data are downloaded as an Excel file from this webserver using a secured link on a secured computer located in a locked room in the Division of Geriatric Medicine of the Jewish General Hospital. There is a double lock system on the computer allowing data access, with separate passwords to access the computer and to open the database. The printed and signed consent forms are also stored in a locked room in the Division of Geriatric Medicine of the Jewish General Hospital, in a locked cabinet. The information collected from this research is not be used for other research purposes and it will be destroyed 10 years following the end of recruited individuals’ participation in this study. If the PI leaves the institution, administrative and ethics rules applicable in Quebec and the CIUSSS West Central Montreal are followed to manage the data. All personal information collected during the study remains confidential within the limits of the law. To protect participant identity, the name and other identifying information are replaced by a code (numerical incremental code), which is the only information about participants to which the PI and representatives have access during the study. Only the project manager is able to link participant codes to participant identities. No information that discloses participant identities is communicated. The coded information may be communicated to Health Canada in the case of an audit. The participants’ coded study information and the participants’ consent forms are also destroyed 10 years following the end of their participation in this study, as consent forms are part of participants’ research file and follow the same rules of recorded data in this study.

### Power calculation

The number of participants required for this study has been calculated to detect the expected change in WEMWBS score between the baseline assessment (M0) and the end of the 3-month cycle (M3) calculated using the formula ((score M^*n* + 1^ – score M^*n*^)/((score M^*n* + 1^ + score M^*n*^)/2)) × 100. The expected difference between intervention and control group was estimated 5.2 ± 10.3 based on a previous study [21]. A free sample size calculator (http://www.sample-size.net/sample-size-means/) was used*.* The minimum number of participants to show an absolute difference between the 2 groups (i.e., intervention and control groups) with alpha = 5% and power = 90% is of 63 per group. Thus, the theoretical total number of participants for the study is 126. Anticipating an 18% rate of participant loss during the follow-up period, the total number of participants required is 150 (75 participants per group).

### Data analysis

Analyses will be performed (1) with the intention to treat for all enrolled participants randomized into two groups (i.e., intervention versus control) and (2) per protocol (i.e., removing data from participants who were not compliant with the protocol) for participants who will be entirely assessed through an 83.3% (i.e., participants who attended 10 on 12 workshops) compliance to the intervention. Analyses will be done through the SPSS Software (version 23.0; SPSS, Inc., Chicago, IL). Missing data will be taken into account with regression imputation. Data will be imputed only if the percentage of participants with missing data is under 10%. The continuous variables’ distributions will be studied (mean, median, mode, minimum, maximum, confidence interval around mean, standard deviation). For the categorical variables, frequencies will be calculated. First, the participants’ baseline characteristics (age, sex, etc.) will be described for each group and compared (i.e., intervention versus control) using parametric and non-parametric tests according to variables’ distribution. Pearson’s chi-square tests (or Fisher’s exact tests) will be used for qualitative variables. For quantitative variables, the statistical tests used will be unpaired *t* tests or non-parametric Mann-Whitney *U* tests for comparing 2 groups, as appropriate.

Second, group comparisons of changes (i.e., ((score M^*n* + 1^ – score M^*n*^)/((score M^*n* + 1^ + score M^*n*^)/2)) × 100) in outcome values between M0 and M1, M0 and M2, M0 and M3, M0 and M9, and M0 and M15 will be performed using repeated measures tests as appropriate. The global significance limit will be set at 0.05 and all tests will be bilateral. In addition to this univariate analysis, a multivariate analysis will be performed to determine the effect of the intervention while adjusting for age, sex, number of medications taken daily, and CPQ score. These adjusted variables are covariates that may influence the relationship between outcome measures (i.e., well-being, quality of life, and frailty) and the participatory art-based activity intervention.

Third, to identify the outcomes with the highest magnitudes of change (i.e., greater effect attributable to the intervention), we will graph the mean difference of each outcome that is significantly different when comparing intervention group participants to those in the control group. Results will be presented as forest plots.

Fourth, analysis of compliance criteria will be based on between-group comparisons using, for quantitative variables, parametric and non-parametric tests according to variables’ distributions, and, for qualitative variables, Pearson Chi-square tests.

An intermediate analysis will be carried out with the first 90 participants. It will focus on the primary evaluation criterion and consider a *p* value < 0.001 sufficiently significant to stop the study.

The final analysis of the primary evaluation criterion in all 150 patients will consider the value *p* < 0.0498 as significant (Haybittle-Peto method).

### Ethical considerations

The Jewish General Hospital (McGill University, Quebec, Canada) Research Ethics Committee approved the study (# 2019-1493).

### Risk to participants

The interventions in our RCT are safe, given the low risk they pose to participants. Indeed, there are no foreseeable risks associated with the participation other than the known rare risks associated with collecting clinical information at each assessment, which could make participants aware of their social and health issues or disabilities. The use of validated, standardized and suitable questionnaires for older adults and the fact that participants can request help and information when filling the questionnaires will minimize these risks. No participants are deprived of any clinically indicated health or social services, and they will continue to be followed by their regular health and social professionals. We estimate that the risk/benefit ratio is low. In addition, to minimize potential risks, the RCT is conducted in compliance with Good Clinical Practices (GCP), providing assurance that the rights, safety, and well-being of participants are protected. The protocol has been submitted to the Jewish General Hospital (McGill University, Quebec, Canada) Research Ethics Committee. A steering committee and an independent 3-member Data and Safety Monitoring Board (DSMB) monitor participant safety. Adverse events are collected during follow-up assessments.

### Steering committee

There is a steering committee composed of a principal investigator, co-investigators, and knowledge users (representative of MMFA and older community dwellers). The roles of the steering committee are the following: (1) to provide advice and ensure the delivery of project outputs and the achievement of outcomes; (2) to make decisions regarding the procedure of the trial, especially its premature discontinuation or continuation and re-assessment of the study power; and (3) to coordinate and validate the communication study results, regardless of the media used.

The steering committee meets at key stages of the RCT: (1) before the submission of the study protocol to the Ethics Committee for correction and validation of the study protocol, (2) between Ethics Committee approval and the baseline assessment, (3) during the follow-up period of the RCT, (4) after the first data analysis, and (5) after the writing of each RCT manuscript, to ensure that agreements are met following intervention, in accordance with the rules of the study protocol and recommendations from the Group of Vancouver.

The steering committee works in partnership with the Data and Safety Monitoring Board (DSMB) which acts in an advisory capacity to independently review the protocol and informed consent forms, monitor the safety of study participants, ensure the confidentiality of RCT data, and evaluate the progress of the RCT.

### Trial status

The study is ongoing. The number of the final protocol version 5 date November 12, 2018, (NCT03679715; Title: A-Health RCT: Effects of Participatory Art-Based Activity on Health of Older Community Dwellers; First posted date: September 20, 2018; prospectively registered; https://clinicaltrials.gov/ct2/show/NCT03679715) and approved by Ethic committee is 2019-1493 dated November 20, 2018. The first participant was recruited on March 4, 2019, and the end of the study is planned for the end of January 2021.

## Discussion

*Art is good for your health* is a frequently encountered mantra in mainstream media, reflecting the growing amount of published evidence regarding art-based activities’ positive effects on health and well-being. Unfortunately, there is a lack of robust data based on gold-standard experimental study designs like RCTs. Furthermore, this advice is applicable to mental health (i.e., well-being and quality of life) in the elderly population but must still be validated for older community dwellers’ physical health condition. Nascent research and action in the field of health prevention and promotion using art-based interventions highlight the key role museums will play as partners in health policy [8, 17–19]. The MMFA is a Canadian leader in this field, partnering with health professionals and researchers. With the A-Health RCT, we have a unique opportunity to demonstrate that an art-based activity like “Thursdays at the Museum” is beneficial to both the mental and physical health of Montreal’s older community dwellers.

The use of art-based activities to improve mental and physical health is not a new concept, as exemplified by the art therapy field’s therapeutic application of art-making [4, 5, 7]. Art therapy is a non-pharmacological approach to improving well-being and quality of life used in patients with cancer, neuropsychiatric diseases, and physical disabilities [7]. In the past decade, the field of art therapy has undergone a shift toward novel application art-based activities, benefiting individuals other than patients with obvious health and wellness needs and expanding settings to include museums [8, 19].

There is growing evidence that art-based activities have positive benefits for patients, such as improvements in self-esteem, confidence, and mood [6, 7]. In addition, these art-based activities are performed in groups, which stimulates social interaction and engagement [7]. Indeed, art-making as part of a group not only engages participants in the creative process directly, but also allows them to become co-authors and observers of others’ work. In contrast, when compared to those performed in patients, there is a limited number of studies showing that participatory art-based activities may improve well-being and/or quality of life either in older adults in residential care facilities or in community-dwelling older adults in relatively good health [8, 19, 20, 27]. Further, these studies reported benefits to well-being and quality of life but did not examine changes in physical health condition. Our previous study, Art-Health (A-Health), showed for the first time that it was possible to act on the physical health condition of older community dwellers through participatory art-based activities, as the proportion of frail participants decreased after a 3-month cycle of “Thursdays at the Museum.” This study introduced the prospect of using art-based activities for health prevention in aging populations at risk for adverse health events. However, this study had two major limitations. First, as with any novel research question, there is a need to show that its results are replicable. Second, its design is not the strongest to demonstrate the effect of an intervention. Thus, A-Health RCT has been launched to overcome these limitations and confirm the initial positive results.

The A-Health RCT study is performed at the MMFA. Museums have opened their doors to those interested in improving health and well-being, broadening their services. They have created programs for different categories of patients and individuals and, thus, have become key partners for health prevention and promotion. “Thursdays at the Museum,” developed by the MMFA, may be assimilated into health prevention and promotion efforts. Acting early in elders’ weakening process in order to keep them in good health and socially active for longer is possible [28]. However, very few museum programs have focused on elders [8, 19, 20, 27], and only the MMFA’s program has focused on elders living with early frailty, demonstrating that it is possible to improve the health status of elders living at home through participatory visual art-based activities. This program originated from the partnership of two significant actors in Montreal: the MMFA and the CEEXLO of the McGill University Integrated Health Network. These partners are developing a program known as the “Arts & Longevity Lab.” Several prefiguring characteristics of living labs underlie the successful cooperation of the MMFA and CEEXLO: (a) the synergetic experiences, skills, and know-how that these two partners consolidate to achieve the common objective of proposing an innovative, validated intervention that meets the expectations and needs of the elderly; (b) the place of experimentation, the MMFA, which has succeeded in expanding its primary function as an exhibition space into a research-friendly place; and (c) the experimental research-action approach, which places elders at the heart of the evaluation process through digital self-evaluations on the CEEXLO platform. Over the past 3 years, this collaboration has been enriched by partnerships from many sectors and disciplines, including: a) researchers from the health, arts and culture, social science and education fields; b) the Arts & Health Committee of the MMFA; c) a private company specialized in digital health.

“Thursdays at the Museum” is a MMFA initiative echoing several World Health Organization (WHO) actions. Indeed, the WHO has argued in favor of proactive and positive approaches to chronic disease management (https://www.who.int/mental_health/neurology/dementia/action_plan_2017_2025/en/, https://www.who.int/ageing/healthy-ageing/en/). The organization proposes a “life course” approach to deal with the health issues associated with aging and recommended implementing programs oriented toward positive interventions. The Aging and Health Program was launched by the WHO in 2015 and included the promotion of health and culture as a key component (https://www.who.int/ageing/healthy-ageing/en/). This program helped to create a much broader approach to health promotion with a strong community focus. Throughout much of the world, healthcare is delivered in clinics and hospitals, while health promotion and illness prevention activities mostly occur in schools, community organizations, or at work. While these are suitable locations that reach a great number of people, there are additional organizations and sectors that could become partners in public health research and practice development. Museums are among such potential partners. They are aware of the needs of their communities and are consequently expanding the types of activities they offer [21, 28, 29].

The A-Health RCT has some limitations. First, participants are not blind to the intervention. Second, there is the possibility of contamination bias, as control group participants may participate in other cultural activities during the intervention period. These participants are therefore requested to withhold participation in activities involving art for the period of the study and to report any other activity practiced during the same period. In addition, since only intervention group participants may partake in “Thursdays at the Museum” activities, contamination bias is limited in our RCT. Each participant also signs an attendance form before the beginning of each workshop for the whole 3-month cycle. Nevertheless, these self-reports cannot guarantee that participants will not engage in activities that can interfere with their well-being, quality of life, or frailty condition. Third, the A-Health RCT is a monocenter study performed in Montreal in older adults who must have access to an Internet connection, which is a limitation to the results’ external validity. However, the increasing use of social technology among older adults (e.g., use of Internet-based e-mail or video calls) highlights the potential of this medium in our population. Fourth, assessments are mainly self-administered questionnaires that may lead to misreporting.

The results of the RCT will be scrutinized at three levels. First, at a scientific level, measuring the effects on well-being, quality of life, and frailty may provide a better understanding of the interactions between these three domains and may thusly be used to develop specific interventions based on the elucidated mechanism. Second, at a policy level, as exemplified by the English Alliance of Museums for Health and Well-being (https://museumsandwell-beingalliance.files.wordpress.com/2018/04/museums-as-spaces-for-well-being-a-second-report.pdf), the results of this RCT may be a source of information and advice to adjust health policy and incorporate museums in efficient and validated interventions. Third, at a global level, the results will also be presented and disseminated within an international consortium of Research-Museum teams, which was created in 2016 created by the PI of the RCT. These presentations will give members of this consortium the opportunity to initiate similar projects, respecting the same procedures and using the formalized support guide, while adapting to local constraints and characteristics. If the project is replicated, it will be possible to merge the resulting data into a single database that will allow for the analysis of new variables and may influence or enrich the primary results and conclusions. Finally, this database will allow researchers from any discipline and country, even if they did not participate in the first project, to use the knowledge acquired for research purposes or to enrich it with new data of their own.

In conclusion, participation in museums’ art-based activities has received growing interest from researchers and policy-makers due to their potentially positive effects on mental and physical health. There are few robust studies like RCTs that focus on older community dwellers and examine the efficacy of these participatory museum activities. The A-Health RCT is an opportunity to confirm a participatory art-based museum activity’s effects on mental and physical health and to show the key role museums can play in health promotion for the elderly population.

## Data Availability

The datasets used and analyzed in the current study will be made available by the corresponding author upon reasonable request. Requests should be sent to the corresponding author: Olivier Beauchet, MD, PhD; Department of Medicine, Division of Geriatric Medicine, Sir Mortimer B. Davis Jewish General Hospital, McGill University, 3755 chemin de la Côte-Sainte-Catherine, Montréal, QC H3T 1E2, Canada; E-mail: olivier.beauchet@mcgill.ca. All requests need a cover letter explaining the objective, justification, and referent ethic committee.

## Abbreviations

MMFA: Montreal Museum of Fine Arts
CEEXLO: Centre of Excellence on Longevity
RUISSS: Integrated University Health and Social Services Network
CPQ: Computer Proficiency Questionnaire
CESAM: Centre of Excellence Self-AdMinistered questionnaire
ADL: Activities of daily living
IADL: Instrumental Activities of Daily Living
EQ-5D: EuroQol-5D
VAS: Visual analogic scale
WEMWBS: Warwick-Edinburgh Mental Well-Being Scale
